# Comparison of chest CT features between progressive and nonprogressive patients with COVID-19 pneumonia: A meta-analysis

**DOI:** 10.1097/MD.0000000000030744

**Published:** 2022-09-30

**Authors:** Haijing Wang, Lin Luo, Wenwu Lv, Tao Jin, Mingkuan Jiang, Miao Miao, Qiang Chen

**Affiliations:** a Baotou Medical College, Inner Mongolia University of Science and Technology, Baotou City, Inner Mongolia Autonomous Region, China; b Department of Imaging, the First Affiliated Hospital of Baotou Medical College, Inner Mongolia University of Science and Technology, Baotou City, Inner Mongolia Autonomous Region, China.

**Keywords:** computed tomography, COVID-19, meta-analysis

## Abstract

The aim of this study was to compare the radiographic features of patients with progressive and nonprogressive coronavirus disease 2019 (COVID-19) pneumonia.

**Methods:**

PubMed, Embase, and Cochrane Library databases were searched from January 1, 2020, to February 28, 2022, by using the keywords: “COVID-19”, “novel Coronavirus”, “2019-novel coronavirus”, “CT”, “radiology” and “imaging”. We summarized the computed tomography manifestations of progressive and nonprogressive COVID-19 pneumonia. The meta-analysis was performed using the Stata statistical software version 16.0.

**Results:**

A total of 10 studies with 1092 patients were included in this analysis. The findings of this meta-analysis indicated that the dominating computed tomography characteristics of progressive patients were a crazy-paving pattern (odds ratio [OR] = 2.10) and patchy shadowing (OR = 1.64). The dominating lesions distribution of progressive patients were bilateral (OR = 11.62), central mixed subpleural (OR = 1.37), and central (OR = 1.36). The other dominating lesions of progressive patients were pleura thickening (OR = 2.13), lymphadenopathy (OR = 1.74), vascular enlargement (OR = 1.39), air bronchogram (OR = 1.29), and pleural effusion (OR = 1.29). Two patterns of lesions showed significant links with the progression of disease: nodule (*P* = .001) and crazy-paving pattern (*P* = .023). Four lesions distribution showed significant links with the progression of disease: bilateral (*P* = .004), right upper lobe (*P* = .003), right middle lobe (*P* = .001), and left upper lobe (*P* = .018).

**Conclusion:**

Nodules, crazy-paving pattern, and/or new lesions in bilateral, upper and middle lobe of right lung, and lower lobe of left lung may indicate disease deterioration. Clinicians should formulate or modify treatment strategies in time according to these specific conditions.

## 1. Introduction

At present, the coronavirus disease 2019 (COVID-19) pandemic is still very severe, and the number of confirmed cases in countries around the world keeps increasing. According to the information provided by the official China Chinese Center for Disease Control and Prevention, as of February 28, 2022, >200 countries around the world have been involved, and a total of 410 million cases have been confirmed cumulatively in the globe, with about 5.8 million deaths. COVID-19 has brought great harm to countries around the world and has posed a serious threat to human life and health.^[1,2]^ Currently, the gold standard for the diagnosis of COVID-19 is still reverse transcription-polymerase chain reaction (RT-PCR) technology.^[3]^ However, it has a very limited part in the evaluation and prediction of severity of disease.^[4]^ Radiology plays an important role in the early detection and management of COVID-19 patients.^[5]^ Among various imaging methods, chest computed tomography (CT) has become the primary screening and dynamic review tool for COVID-19 due to its high sensitivity and specificity.^[6]^ Several studies have compared the sensitivity of CT and RT-PCR for the diagnosis of COVID-19; the results showed that for the patients with the first RT-PCR and CT examination, the sensitivity of CT examination for the diagnosis of COVID-19 was >95%, which was significantly higher than the sensitivity of RT-PCR.^[7–10]^ Moreover, CT scan can detect lesions early, observe the scope of the abnormalities, assess the severity of the lesions, and assist the clinician in completing rapid isolation, diagnosis, and treatment. CT imaging played an important role in controlling the epidemic situation in China.^[11]^ Moreover, CT imaging can help to understand the performance of COVID-19 in different stages and dynamically detect changes in the patient’s condition.^[12]^ The CT findings are closely related to the time course and show different imaging findings with progression.^[13]^ Our study aims to compare the radiological characteristics of progressive and nonprogressive patients with COVID-19 pneumonia and further integrate the imaging information to provide clues for the clinical diagnosis and treatment. This article is written with reference to the PRISMA statement.^[14]^

## 2. Materials and Methods

### 2.1. Search strategy

This study was approved by the Institutional Review Board and Ethics Committees of Baotou Medical College. Written informed consent was waived for the retrospective analyses by the Institutional Review Board. We searched literature on PubMed, Embase, and Cochrane Library databases from January 1, 2020, to February 28, 2022. The literature retrieval used a combination of subject words and free words and adjusted according to different database characteristics. Search keywords are (“Novel Coronavirus” OR “COVID-19” OR “2019-nCoV” OR “New Coronavirus” OR “2019-Novel Coronavirus” OR “Corona Virus Disease” OR “SARS-CoV-2” OR “Coronavirus Disease” OR “COVID-2019”) AND (“CT” OR “Radiology” OR “Radiologic” OR “Imaging” OR “Computed Tomographic” OR “Radiological” OR “Radiologist” OR “Computed Tomography” OR “Radiographic”). In addition, we checked the reference lists of all key articles for any additional eligible articles.

### 2.2. The definition of progressive and nonprogressive patients with COVID-19

**Figure 1.**
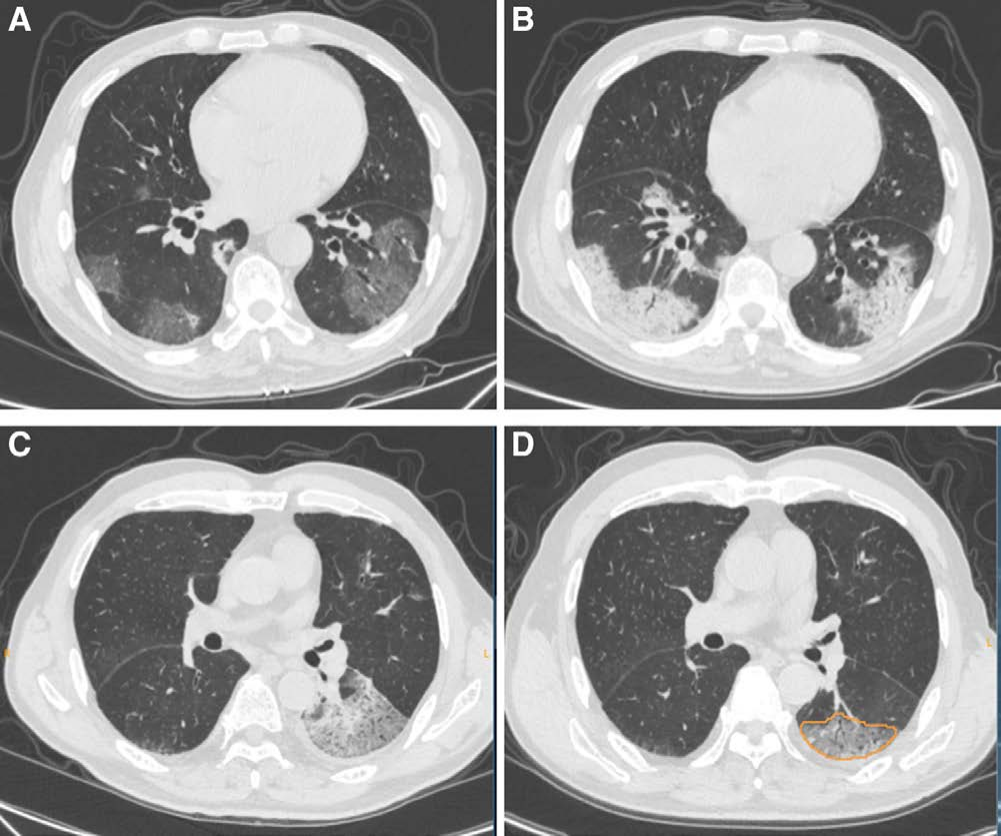
Typical CT images of progressive and nonprogressive patients with COVID-19. (A, B) A 53-yr-old male patient with COVID-19 in the progression group. Axial CT image (A) from the initial scan shows the round and patchy GGO distributed around the bilateral lower lobes. Axial image (B) from the follow-up CT obtained 4 d later shows that the density and size of GGO were increased, and the consolidation was significantly increased. (C, D) A 45-yr-old male patient with COVID-19 in the nonprogression group. Axial CT image (C) from the initial scan shows the patchy GGO and consolidation distributed around the left lower lobes. Axial image (D) from the follow-up CT obtained 5 d later shows that the density and size of GGO were reduced, and the consolidation was disappeared. COVID-19 = coronavirus disease 2019, CT = computed tomography, GGO = ground glass opacity.

The progression group included those COVID-19 patients showing an increase in size and/or density of lesions, while the nonprogression group included those who showed a reduction or no change in size and/or density of lesions.^[15]^ CT images to illustrate the differences between progressive and nonprogressive patients are shown in Figure 1.

### 2.3. Selection criteria

The inclusion criteria for the meta-analysis were as follows: (a) studies on patients with RT-PCR examination confirmed COVID-19 pneumonia; (b) studies reported and compared CT characteristics between progressive and nonprogressive patients with COVID-19 pneumonia; (c) the study type was observational studies, including cohort studies, case series studies, and case–control studies; and (d) the language is English.

The exclusion criteria were as follows: (a) reviews, letters, comments, and guidelines; (b) those studies irrelevant to the subject of the present study; (c) researches focus on special groups, such as research on family cluster cases, children, the elderly, pregnant women, and medical workers; (d) the number of COVID-19 patients included in the literature was <20; and (e) full-text literature is not available.

### 2.4. Data extraction

Literature titles, abstracts, and full text were screened by 2 investigators (L.L. with 11 years of clinical experience; and M.K.J. with 5 years of clinical experience) independently based on the inclusion and exclusion criteria of the literature. The process of screening was performed under the Endnote X9 software. After we determined the inclusion of associated literature, we extracted the following variables: name of the first author, publication year, research type, experimental design, number of patients, and case source.

Chest CT features of COVID-19 pneumonia patients were summarized into 3 aspects: pattern of lesions, lesions distribution, and pleural, bronchial, vascular, and mediastinal lesions. A total of 7 patterns of lesions including ground glass opacity (GGO), consolidation, GGO mixed consolidation, nodule, crazy-paving pattern, patchy shadowing, and halo sign were extracted. The variables extracted from the distribution of lesions included the following 10 aspects: right upper lobe, right middle lobe, right lower lobe, left upper lobe, left lower lobe, central, subpleural, both central and subpleural, unilateral, bilateral. The following 6 variables were mainly extracted from pleural, bronchial, vascular, and mediastinal lesions, including air bronchogram, pleural thickening, bronchodilation, pleural effusion, lymphadenopathy, and vascular enlargement.

### 2.5. Quanlity evaluation of included studies

All included literature were evaluated using the Newcastle–Ottawa Scale,^[16]^ which consists of 3 factors: object selection, comparability of the study groups, and assessment of outcome. A score of 0 to 9 (allocated as stars) was allocated to each study. Studies with a score of >6 were considered as high quality. Quality evaluation was performed independently by 2 reviewers (W.W.L. with 9 years of clinical experience; and M.M. with 5 years of clinical experience). If there is a disagreement, consensus was reached through discussion with the third reviewer (Q.C. with 13 years of clinical experience).

### 2.6. Statistical analysis

Statistical analysis was performed using the STATA version 16.0 software. The association between the CT features and the progression of COVID-19 pneumonia was assessed in the form of odds ratio (OR) at a 95% confidence interval (95% CI). Heterogeneity between the studies was assessed using Cochran Q test and Inconsistency index (*I*^2^) test.^[17]^ When *I*^2^ was <50%, a fixed-effects (Mantel–Haenszel model) model was used for the analysis, otherwise, a random-effect model was adopted. Subgroup analysis was performed for case source (inside and outside Hubei Province) and experimental design (single-center and multicenter studies) to explore the sources of heterogeneity. Publication bias was detected by Begg rank correlation method and funnel plot. When the funnel plot is dissymmetrical or statistic Z > 1.96 and *P *< .05 calculated by Begg rank correlation method, it suggests publication bias. Bilateral *P* < .05 was considered statistically significant.

## 3. Results

### 3.1. Literature retrieval

**Figure 2.**
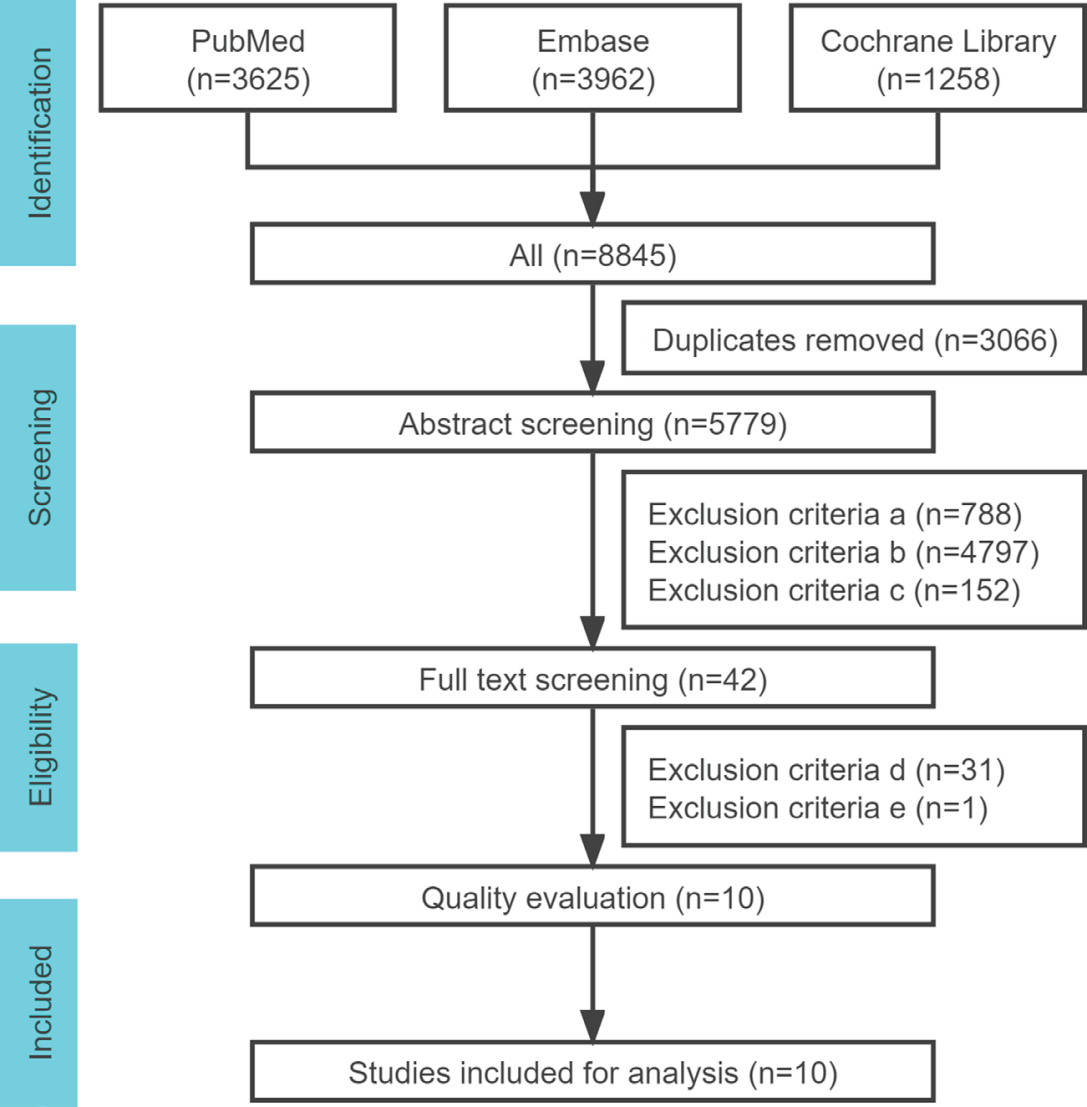
Flowchart of the process of identifying relevant studies.

From the databases mentioned above, we retrieved 8845 articles. After removing 3066 duplicated articles, 5779 articles remained. After reading the titles and abstracts, a total of 5736 articles meeting the exclusion criteria a, b, c were excluded. The remaining 42 articles were further processed for full-text reading, and a total of 32 articles meeting the exclusion criteria d and e were exclude. Finally, we kept 10 studies including 1092 COVID-19 pneumonia patients in this meta-analysis. The specific procedure is shown in Figure 2.

### 3.2. Characteristics of included literature and quality evaluation

In 10 included studies, 9 of them are case–control studies, another one is case series study; 8 of them are single-center studies, the others are multicenter studies; 3 of them come from Hubei Province, 6 of them come from outside of Hubei Province, another one comes from both inside and outside of Hubei province, China. According to the results of quality evaluation of the included literature, all the literature were scored between 7 and 9 points (stars), indicating that the included literature were of good quality. The primary characteristics of the literature are summarized in Table 1.

**Table 1:** Characteristics of the included literatures

First author	Year	Progressive patients	Nonprogressive patients	City/province^a^	Research type	Experimental design	Literature quality^b^
Guan^[17]^	2020	42	12	Beijing	CC	S	8
Li^[18]^	2020	41	50	Chongqing	CC	M	8
Liu^[19]^	2020	24	24	Changsha/Hunan	CS	M	9
Luo^[20]^	2020	15	80	Changsha/Hunan	CC	S	9
Niu^[21]^	2021	237	124	Wuhan/Hubei	CC	S	9
Tan^[22]^	2020	65	95	Wenzhou/Zhejiang, Wuhan/Hubei	CC	S	7
Zhao^[23]^	2020	45	73	Changsha/Hunan	CC	S	8
Zhuang^[24]^	2020	22	22	Wuhan/Hubei	CC	S	8
Zhou^[25]^	2020	28	34	Chongqing	CC	S	8
Zhou^[26]^	2020	22	40	Wuhan/Hubei	CC	S	9

a All included literatures come from China, Beijing, and Chongqing are municipalities directly under the central government.

b Newcastle–Ottawa Scale (NOS) is used for literature quality evaluation

CC = case control study, CS = case series study, M =multicenter study, S = single-center study.

GGO and consolidation were the most common reported CT findings, which were shown in all 10 studies.^[15,18–26]^ The next common CT feature was nodular, shown in 7 studies^[18–23,25]^; GGO mixed consolidation^[18,19,22,23,25]^ and crazy-paving pattern^[17,19,20,24,25]^ were reported separately in 5 studies; patchy shadowing^[17, 19, 20, 22]^ and halo sign^[20,25]^ was displayed in 4 and 2 studies, respectively. Center and subpleural appeared with most frequency in the distribution of lesions, shown in 6 studies^[19,20,22–25]^; both center and subpleural were reported in 5 studies^[19,22–25]^; unilateral and bilateral were shown in 3 studies.^[19,20,23]^ In terms of the position of affected lobes, right upper lobe, right middle lobe, right lower lobe bronchial, left upper lobe, and left lower lobe were all reported in 2 studies.^[17,24]^ In the light of pleural lesions, pleural effusion and pleural thickening appeared in 6 studies^[20,22–26]^ and 2 studies,^[19,26]^ respectively. Moreover, air bronchogram was shown in 7 studies,^[17–20,22,25,26]^ lymphadenopathy was reported in 4 studies,^[20,22,23,25]^ bronchodilation was reported in 3 studies,^[19,22,23]^ and vascular enlargement was reported in 2 studies.^[23,26]^

### 3.3. Meta-analysis

#### 3.3.1. Pattern of lesions.

**Figure 3.**
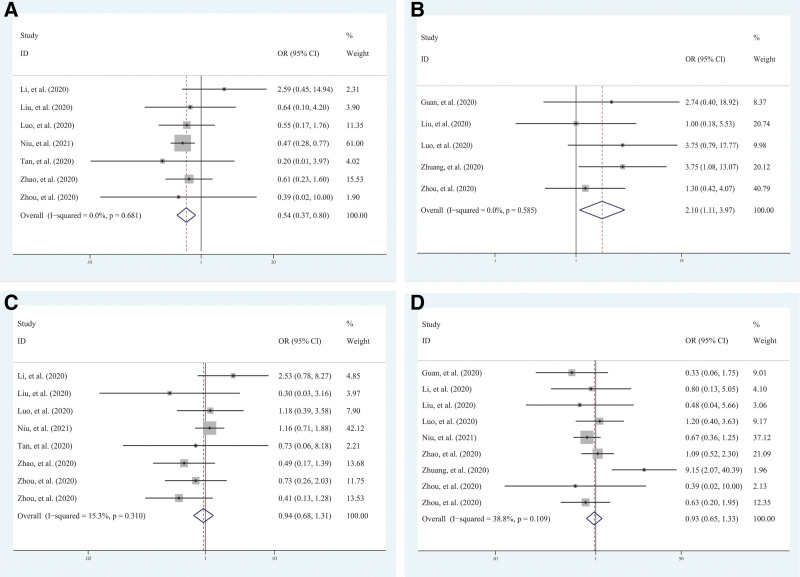
Forest map based on Pattern of lesions. Forest plots in the meta-analysis based on nodule (A), crazy-paving pattern (B), GGO (C) and consolidation (D). The ORs with 95% CIs were calculated using fixed-effects model. GGO = ground glass opacity, OR = odds ratio.

Compared with the nonprogressive patients, the findings of this meta-analysis indicated that the predominant CT features of the progressive patients were crazy-paving pattern and patchy shadowing. Nodule and halo sign were relatively rare in the progressive patients. Two CT features showed significant links with the progression of disease: nodule (OR = 0.53; 95% CI = 0.36–0.78; *P* = .001) and crazy-paving pattern (OR = 2.10; 95% CI = 1.11–3.97; *P* = .023) (Fig. 3). The remaining 5 features did not exhibit an apparent association with the progression of disease: GGO (OR = 0.94; 95% CI = 0.68–1.31, *P* = .714), consolidation (OR = 0.93; 95% CI = 0.65–1.33, *P* = .685), GGO mixed consolidation (OR = 0.81; 95% CI = 0.52–1.25, *P* = .334), patchy shadowing (OR = 1.64; 95% CI = 0.90–2.99, *P* = .109), and halo sign (OR = 0.29; 95% CI = 0.04–2.00, *P* = .209). All these data are illustrated in Table 2.

**Table 2:** CT imaging characteristics in progressive and nonprogressive patients with COVID-19.

Variable	No. of included studies	Patients (n) PP/NPP	OR (95%CI)	*P* value	*P*_*h*_	*I*^2^ (%)
Pattern of lesions						
GGO	10	539/553	0.94 (0.68–1.31)	.714	.310	15.3
Consolidation	10	539/553	0.93 (0.65–1.33)	.685	.109	38.8
Both GGO and consolidation	5	203/276	0.81 (0.52–1.25)	.334	.140	42.2
Nodule	7	455/480	0.53 (0.36–0.78)	.001	.681	0
Crazy-paving pattern	5	129/171	2.10 (1.11–3.97)	.023	.585	0
Patchy shadowing	4	144/210	1.64 (0.90–2.99)	.109	.204	34.7
Halo sign	2	43/114	0.29 (0.04–2.00)	.209	.178	44.8
Lesions distribution						
Right upper lobe	2	62/33	5.18 (1.78–15.09)	.003	.636	0
Right middle lobe	2	62/33	5.93 (1.98–17.79)	.001	.581	0
Right lower lobe	2	62/33	2.10 (0.26–17.13)	.490	.048	74.4
Left upper lobe	2	62/33	3.10 (1.22–7.90)	.018	.248	25.2
Left lower lobe	2	62/33	1.60 (0.59–4.34)	.358	.750	0
Central	6	199/328	1.36 (0.53–3.48)	.524	.477	0
Subpleural	6	199/328	0.66 (0.41–1.06)	.085	.587	0
Both	5	184/248	1.37 (0.82–2.29)	.237	.094	49.5
Unilateral	3	84/177	0.45 (0.18–1.13)	.088	.582	0
Bilateral	3	84/177	11.62 (2.17–62.16)	.004	.780	0
Pleural, bronchial, vascular, and mediastinal lesions						
Air bronchogram	7	235/334	1.29 (0.57–2.89)	.541	.011	63.8
Pleura thickening	2	46/64	2.13 (0.97–4.66)	.059	.813	0
Bronchodilation	3	134/192	0.90 (0.16–5.05)	.905	.001	87.4
Pleural effusion	6	197/344	1.29 (0.36–4.58)	.695	.032	59.1
Lymphadenopathy	4	153/282	1.74 (0.40–7.59)	.458	.846	0
Vascular enlargement	2	67/113	1.39 (0.67–2.86)	.425	.302	6.3

CI = confidence interval, COVID-19 = coronavirus disease 2019, GGO = ground glass opacity, NPP = nonprogressive patients, OR = odds ratio, PP = progressive patients.

#### 3.3.2. Lesions distribution.

**Figure 4.**
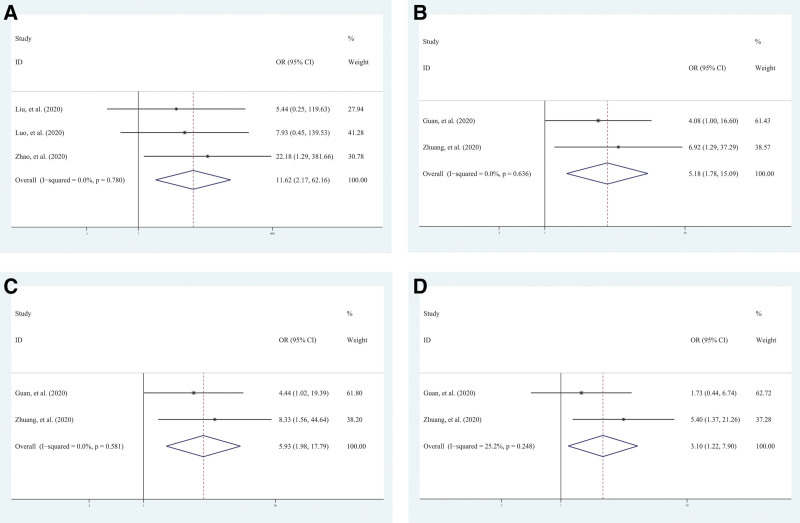
Forest map based on lesion distribution. Forest plots in the meta-analysis based on bilateral (A), right upper lobe (B), right middle lobe (C), and left upper lobe (D). The ORs with 95% CIs were calculated using fixed-effects model. CI = confidence interval, OR = odds ratio.

Compared with the nonprogressive patients, progressive patients were more frequent to show abnormalities at the following locations: bilateral, right middle lobe, right upper lobe, left upper lobe, right lower lobe, left lower lobe, both central and subpleural, and central. On the contrary, progressive patients were less frequent to show unilateral and subpleural pneumonia (Table 2). Four lesions distribution showed significant links with the progression of disease: bilateral (OR = 11.62; 95% CI = 2.17–62.16, *P* = .004), right upper lobe (OR = 5.06; 95% CI = 1.73–14.90, *P* = .003), right middle lobe (OR = 5.93; 95% CI = 1.98–17.79, *P* = .001), and left upper lobe (OR = 3.10; 95% CI = 1.22–7.90, *P* = .018; Table 2; Fig. 4). The remaining 6 variables related to abnormalities locations did not show a significant association with the progression of disease (*P* > .05 for all).

#### 3.3.3. Pleural, bronchial, vascular, and mediastinal lesions.

As shown in Table 2, compared with the nonprogressive patients, the dominating lesions of progressive patients were pleura thickening (OR = 2.13; 95% CI = 0.97–4.66, *P* = .059), lymphadenopathy (OR = 1.74; 95% CI = 0.40–7.59, *P* = .458), vascular enlargement (OR = 1.39; 95% CI = 0.67–2.86, *P* = .425) air bronchogram (OR = 1.29; 95% CI = 0.57–2.89, *P* = .541), and pleural effusion (OR = 1.29; 95% CI = 0.36–4.58, *P* = .695). Bronchodilation (OR = 0.90; 95% CI = 0.16–5.05, *P* = .905) was relatively infrequent in the progressive patients. However, there were no significant links between the above-mentioned lesions with the progression of disease.

#### 3.3.4. Publication bias and subgroup analysis.

**Figure 5.**
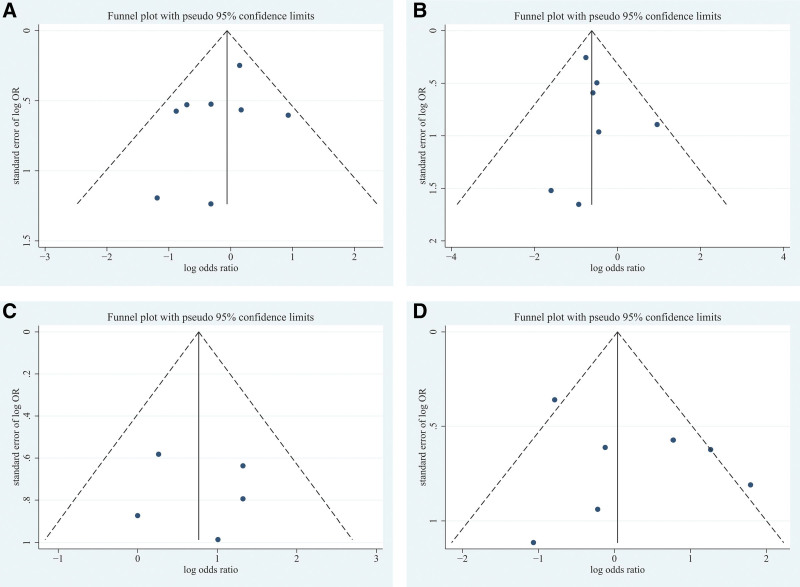
Funnel graph based on the part of the variable. Funnel graph in the meta-analysis based on GGO (A), nodule (B), crazy-paving pattern (C), and air bronchogram (D). Begg rank correlation test showed that all selected variables met Z < 1.96, *P* > .05. Neither the pattern of funnel plot nor the results of the Begg test, there was no publication bias. GGO = ground glass opacity.

Publication bias was tested for the CT features of GGO, nodule, crazy-paving pattern, and air brochogram. It turned out that the funnel plot was roughly symmetrical (Fig. 5). Begg rank correlation test showed that all selected variables met Z < 1.96, *P *> .05 (Z = 0.12, *P *= .902; Z = 0, *P *= 1.000; Z = 0.38, *P *= .707; Z = 0, *P *= 1.000 for GGO, nodule, crazy-paving pattern and air bronchogram, respectively). According to the pattern of funnel plot and the results of Begg test, there was no publication bias.

By subgroup analysis of experimental design for air bronchogram (the heterogeneity was 63.8%), the *I*^2^ value for the single-center study subgroup and multicenter study subgroup were 67.1% and 42.4%, respectively, and there was no significant reduction in heterogeneity. By subgroup analysis of case source for pleural effusion (the heterogeneity was 59.1%), the *I*^2^ value for the inner and outer of Hubei province subgroup were all 0%, and heterogeneity was significantly reduced. Meta-regression analysis revealed that differences in case source could explain 100% of the heterogeneity.

## 4. Discussion

In the 21st century, humans have experienced 3 deadly coronavirus pandemics: severe acute respiratory syndrome (SARS), Middle East Respiratory Syndrome (MERS), and COVID-19.^[2]^ COVID-19 is the third major coronavirus outbreak, which proved to be the most serious among all the previous outbreaks, because it has brought more infections and deaths to a wider range.^[27]^ Coronaviruses are single-stranded RNA viruses and are prevalent in many mammals including humans. Coronaviruses are highly contagious in nature and have caused high mortalities.^[28]^ This kind of virus belongs to the genus Betacoronavirus and subgenus Sarbecovirus and is characterized by the presence of club-shaped spike projections originating from the surface of the virus.^[29]^ According to literature reports, the symptoms are dominantly fever, fatigue, and dry cough, and can be complicated with tiredness, sore throat, and headache. A few patients have symptoms such as stuffy nose, runny nose, and diarrhea.^[30]^ The main transmission routes of COVID-19 from human to human are direct transmission, aerosol transmission, and contact transmission. The rapid infection and high incidence contribute to far-reaching implications for public health and pose a serious threat to human life and health. The diagnosis of COVID-19 relies on positive detection of nucleic acid testing; because of the high specificity but poor sensitivity of nucleic acid test, false negative cases are frequently reported.^[8]^ Thus, the imaging characteristics of COVID-19 are crucial in terms of the early identification, differential diagnosis, as well as subsequent management strategies. Chest CT may be able to detect the disease prior to the development of clinical symptoms. Moreover, chest CT plays an important role in the overall course of COVID-19; it can not only diagnose diseases, but also help clinicians detect dynamic changes and abnormalities in the lungs in time, and further assist physicians in developing treatment strategies.

We performed a meta-analysis of 10 studies involving 1092 patients to provide comprehensive information on chest CT features associated with imaging progression of COVID-19. Compared with the nonprogressive patients, crazy-paving pattern and patchy shadowing were the most common findings in progressive patients. In progressive patients with COVID-19 pneumonia, the lesions distribution is mainly bilateral, central and subpleural, and central. The lesions of pleura thickening, lymphadenopathy, vascular enlargement, air bronchogram, and pleural effusion were more likely to occur in progressive patients. GGO, consolidation, GGO mixed consolidation, nodule, and halo sign in lesions pattern, unilateral and subpleural in the lesions distribution, and bronchodilation were more likely to occur in nonprogressive patients with COVID-19 pneumonia. The results of the meta-analysis suggest that nodule and crazy-paving pattern in pattern of lesions showed significant links with progression with COVID-19 pneumonia. Bilateral, right upper lobe, right middle lobe, and left upper lobe in lesions distribution showed significant links with progression with COVID-19 pneumonia. Pleural, bronchial, vascular, and mediastinal lesions were not significantly associated with disease progression. When chest CT shows nodules, crazy-paving pattern, and/or new lesions in bilateral, upper and middle lobe of right lung, and lower lobe of left lung, it may indicate disease deterioration, and clinicians should develop or modify treatment strategies.

COVID-19 pneumonia should be distinguished from other viral pneumonia, such as SARS, MERS, influenza virus, adenovirus, RSV, etc.^[31,32]^ Compared with COVID-19, H1N1 pneumonia is typically characterized by peribronchovascular or subpleural scattered GGO or consolidation. In H1N1 pneumonia, some patterns, including lymphadenopathy and pleural effusion, are usually absent.^[33]^ For H7N9 pneumonia, the most common findings on CT are GGO, which usually progresses rapidly and the right lower lobe is easier to be involved.^[34]^ The most common radiographic abnormalities in adenovirus pneumonia are consolidation and the likelihood of involvement of bilateral lobes is high.^[35]^ In the RSV-infected patients, the most characteristic signs in chest CT scans are at the beginning of pneumonia with nodules and tree-in-bud often combined with bronchial wall thickening. Compared with other viral pneumonia, conolidation and GGO are rarely observed in RSV-infected pneumonia.^[36]^ SARS, MERS, and COVID-19 viruses all belong to the coronaviruses family; the fatality rate of coronavirus MERS is higher than SARS and COVID-19. However, COVID-19 transmits rapidly in comparison to SARS and MERS.^[37]^ They have many similarities in their CT findings. Imaging findings in early stage of these 3 coronavirus pneumonia show similar basic lesion patterns, including GGO and consolidation, bilateral distribution, and predominant involvement of the subpleural area and the lower lobes. Early fibrotic changes only appear in SARS, and MERS has more severe inflammatory changes, including cavitation and pleural effusion.^[38]^ The final diagnosis is determined by real-time PCR.

The results of this meta-analysis involved 23 indicators. The study showed that the heterogeneity of 18 indicators was small (*I*^2^ < 50%); a fixed-effects (Mantel–Haenszel model) model was used. The heterogeneity of 5 indicators was large (*I*^2^ > 50%), including halo sign, right lower lobe, air bronchogram, bronchodilation, and pleural effusion; the random effect model was adopted. Using air bronchogram and pleural effusion as the observation index, subgroup analysis was conducted on experimental design and source of cases. By subgroup analysis of experimental design for air bronchogram, there was no significant reduction in heterogeneity. By subgroup analysis of case source for pleural effusion, heterogeneity was significantly reduced, and meta-regression analysis revealed that differences in case source could explain 100% of the heterogeneity. Therefore, it can be considered that the case source is the main source of heterogeneity.

To the best of our knowledge, this meta-analysis is the first one that evaluated chest CT features associated with progression of COVID-19 infection. The literature included in this study is of good quality. However, there are also some limitations to this study. On the one hand, only 10 related literature were included in the present study, and the number of literature involving in certain indicator is even small, which limits the strength of the argumentation of the results. On the other hand, all the patients included are Chinese and the conclusions may be less representative.

In a word, our results show that progressive patients are more likely to have CT abnormalities with nodule and crazy-paving patter and are more likely to distribute in bilateral, upper and middle lobe of right lung, and lower lobe of left lung. However, due to the above-mentioned limitations, studies with a larger sample size and a more rigorous design are needed.

## Author contributions

Haijing Wang performed literature research, gathered and analyzed information and wrote the original manuscript. Miao Miao, Mingkuan Jiang and Tao Jin gathered and analyzed information. LinLuo and Wenwu Lv provided research insight content examination. QiangChen completed critical reading, and editing. All authors read and approved the final manuscript.
